# Delayed Symptom Onset Following Pediatric Sport-Related Concussion

**DOI:** 10.3389/fneur.2020.00220

**Published:** 2020-04-03

**Authors:** Ashley Olson, Michael J. Ellis, Erin Selci, Kelly Russell

**Affiliations:** ^1^Max Rady College of Medicine Sciences, University of Manitoba, Winnipeg, MB, Canada; ^2^Department of Surgery, University of Manitoba, Winnipeg, MB, Canada; ^3^Pediatrics and Child Health, University of Manitoba, Winnipeg, MB, Canada; ^4^Section of Neurosurgery, University of Manitoba, Winnipeg, MB, Canada; ^5^Pan Am Concussion Program, Winnipeg, MB, Canada; ^6^Childrens Hospital Research Institute of Manitoba, Winnipeg, MB, Canada; ^7^Canada North Concussion Network, Winnipeg, MB, Canada

**Keywords:** sports-related concussion, pediatric, symptom onset, delayed symptoms, clinical outcomes

## Abstract

**Objective:** (1) To examine the prevalence of delayed symptom onset (DSO) among pediatric sport-related concussion (SRC) patients as well as the effect of symptom onset on initial symptom severity, length of recovery, and development of delayed recovery; (2) to evaluate the impact of symptom onset on sideline management.

**Methods:** We conducted a prospective study of pediatric SRC patients (<20 years of age) evaluated at a multi-disciplinary concussion program. Patients underwent initial medical assessment by a single neurosurgeon and a structured interview by a research assistant. Patients were classified as experiencing early symptom onset (symptom onset <15 min from the time of the suspected injury; ESO) or DSO (≥15 min from the time of the suspected injury).

**Results:** A total of 144 SRC patients (61.1% male; mean age 14.6 years, SD 1.8) evaluated a median of 5.0 days (IQR 4.0, 9.0) post-injury were included in the study. Among these patients, 120 (83.3%) reported experiencing ESO while 24 (16.7%) experienced DSO following injury. Among those that experienced DSO the median length of time from the suspected injury to symptom onset was 60.0 min (IQR 20.0, 720.0). No significant differences were observed in symptom severity at initial medical assessment (median Post-Concussion Symptom Scale score 20.0 vs. 18.0, *p* = 0.35), length of physician-document clinical recovery (median 22.0 vs. 24.0 days; *p* = 0.46) or the proportion of those who developed delayed physician-documented clinical recovery (34.4 vs. 42.1%, *p* = 0.52) among patients with ESO or DSO. Patients who reported experiencing ESO were significantly more likely to be immediately removed from play at the time of their suspected injury compared to those who experienced DSO (71.6% vs. 29.2%; *p* < 0.0001).

**Conclusions:** This study suggests that an important proportion of children and adolescents who sustain an acute SRC experience DSO. DSO is associated with lower rates of immediate removal from play at the time of suspected injury. Secondary study findings highlight the need for improved sport stakeholder concussion education and standardized concussion protocols that mandate the immediate and permanent removal of all youth with a suspected concussion until they undergo medical assessment.

## Background

Concussion is a condition caused by the transmission of biomechanical forces to the brain leading to temporary impairments in neurological functioning that often resolve spontaneously (1). Advanced laboratory and neuro-imaging studies suggest that concussion is associated with acute alterations in cellular metabolism, white matter tract injury, and disruptions in cerebral blood flow regulation that may confer an increased risk to additional trauma (2). Athletes who sustain an acute concussion and continue to participate in sports are at risk of sustaining additional injuries that may be associated with more severe or prolonged symptoms or in rare cases, fatal or disabling injuries related to second impact syndrome or diffuse cerebral edema (3, 4).

In order to prevent these adverse outcomes, current national, and international guidelines recommend that all youth athletes who sustain a suspected concussion during a sporting activity be immediately removed from play and undergo standardized sideline assessment by a licensed healthcare professional (1, 5–7). Where licensed healthcare professionals are not available, urgent referral to a physician for medical assessment is recommended (1). Despite the development of additional sideline screening tools and standardized concussion protocols, proper recognition, and removal of athletes with suspected concussion continues to remain a challenge, and is highly dependent on the athlete's timely recognition and reporting of their own symptoms. Although previous studies have examined clinical outcomes among athletes who continued to play despite a symptomatic sport-related concussion (SRC) (8, 9), little research attention has been paid to athletes who experience delayed symptom onset (DSO) and may be at risk of additional injury during continued sport participation (10–12). One prospective study among collegiate athletes found that athletes who underwent delayed removal from play were more likely to have reported DSO (13). To date, no prospective studies have examined the prevalence of DSO among children and adolescents with acute SRC and its effect on clinical outcomes and athlete sideline management.

Therefore, the primary objectives of this study were to examine the prevalence of DSO among pediatric SRC patients referred to a multi-disciplinary concussion program and evaluate the effect of self-reported symptom onset on clinical outcomes such as initial symptom severity, length of clinical recovery, and risk of delayed clinical recovery. The secondary objective was to describe patients' symptom onset, sideline assessment and management after their concussion.

## Methods

### Study Design and Recruitment

Pediatric SRC patients included in this study were prospectively recruited from the Pan Am Concussion Program in Winnipeg, Manitoba. The Pan Am Concussion Program is a government-funded, multi-disciplinary pediatric concussion clinic that receives referrals for acute SRC patients from primary care, sports, and emergency medicine physicians, and through standardized sport-specific concussion protocols (11). Inclusion criteria for the study included: (1) age 19 years or younger; (2) physician diagnosis of concussion; (3) injury occurring during a sport or recreational activity; and, 4) symptomatic at the time of initial assessment. Exclusion criteria included: (1) no definitive injury date; (2) self-reported symptom onset >1 week from the date of suspected injury; (3) diagnosed with persistent post-concussion symptoms (symptoms >28 days) at initial visit; and, (4) patients who reported transient non-specific symptoms during sporting activities but who did not meet the clinical criteria for concussion as assessed by the physician. Following initial medical assessment by a neurosurgeon, eligible patients and their parents underwent informed assent and consent, respectively, by a research assistant. Afterwards, consented patients completed a structured 20-min oral interview that included open and closed-ended questions that allowed researchers to collect details regarding the mechanism of injury, symptom onset, whether, and how timely they were removed from play, how truthful they were during sideline assessment, whether they returned to play, and if they sustained additional instances of body or head contact. The research assistant recorded the responses on the structured data collection form. In the event that a sideline assessment was performed, the treating physician and research assistants did not have access to this data. No data was collected from sideline assessments provided by sideline healthcare providers (e.g., athletic therapists). The study was approved by the University of Manitoba Bannatyne Research Ethics Board.

### Clinical Assessment and Management

At the time of initial medical assessment at the concussion program, all patients completed a standardized data collection form that included demographic data, past medical history, and the Post-Concussion Symptom Scale (PCSS), a valid and reliable post-concussion symptom inventory (14, 15). All patients subsequently underwent assessment by a single neurosurgeon including a clinical history and physical examination that included evaluation of cranial nerve, motor, sensory, reflex, cerebellar, balance, vestibulo-ocular, and cervical spine functioning. Following medical diagnosis of SRC, patients were seen in follow-up on a weekly basis or as dictated by their rate of recovery and sport schedules. In general, patients met the criteria for physician-documented clinical recovery when they were asymptomatic or back to their pre-injury neurological status at rest, were tolerating full-time school without symptoms, and had a normal physical examination including no evidence of vestibulo-ocular or cervical spine dysfunction (16, 17). Patients were also required to complete the International Consensus on Concussion in Sport graduated Return-to-Play protocol that was specific to their sport where applicable (18). Based on the discretion of the neurosurgeon, some patients underwent graded aerobic treadmill testing and/or neuropsychological testing to confirm clinical recovery. These supplemental tests were considered in patients with more complex medical histories and pre-existing conditions that present with concussion-like symptoms (e.g., migraine, mental health disorders, previous concussions) and those returning to sports with a higher risk of concussion (e.g., hockey, football).

### Definitions and Outcome Measures

The diagnosis of SRC was made by the neurosurgeon based on definition set forth by the International Consensus on Concussion in Sport (18). SRC was diagnosed in patients who reported sustaining a traumatic force or blow to the head, neck, face, or body and presented with new concussion-like symptoms identified on clinical interview or the PCSS including symptoms such as headaches, sensitivity to light or sound, dizziness, or difficulty with remembering or concentrating that could not be attributed to a more appropriate medical diagnosis.

At the time of this study, the authors were not aware of any standardized definitions for delayed symptom onset (DSO) following SRC. Therefore, for the purposes of this study we defined DSO as the onset of initial self-reported concussion-like symptoms ≥15 min from the time of the suspected injury. Patients who experienced the onset of concussion-like symptoms <15 min from the time of the suspected injury were classified as those with early symptom onset (ESO). Fifteen minutes was chosen as a cut-off time point because this is the estimated amount of time it would take for a licensed healthcare professional to remove an athlete with a suspected concussion from play and perform a sideline assessment that does or does not include the use of the Sport Concussion Assessment Tool 5 (SCAT5) or Child SCAT5 (19).

Length of physician-documented clinical recovery was defined as the time from the date of injury to the date of the final follow-up visit in which clinical recovery was documented by the treating neurosurgeon. Because the majority of pediatric concussion patients recover within 1 month, delayed physician-document clinical recovery was defined as a patient who achieved physician-documented clinical recovery >28 days post-injury (20).

### Data Analysis

Normally distributed data were summarized as means and standard deviations and were compared using an unpaired *t*-test. Skewed continuous data were reported as the median with the interquartile range (IQR) and were compared using the rank sum test. Proportions were calculated for dichotomous or polychotomous data and differences were compared using a chi squared test or Fisher's exact test as appropriate. A *p* < 0.05 was defined as statistically significant.

A sensitivity analysis was performed to determine if there were any systematic differences in baseline characteristics between those patients who were followed to physician-documented clinical recovery and those who were lost to follow-up. A second sensitivity analysis was conducted to look at time to recovery or lost to follow-up by symptom onset status.

## Results

From September 2016 to April 2018, 152 SRC patients were approached to participate in the study; however, eight were excluded due to non-sport related mechanisms of injury (*n* = 2), clinically asymptomatic at time of initial assessment (*n* = 2), no definitive injury date (*n* = 2), presenting with PPCS (*n* = 1), and self-report of symptom onset more than 1 week from the time of suspected injury (*n* = 1). No patient who met study eligibility and was approached declined to participate.

Overall, 144 SRC patients (61.1% male; mean age 14.6 years, SD 1.8) were included in the study. The median time from injury to initial medical assessment at the concussion program was 5.0 days (IQR 4.0, 9.0) and the median PCSS score at initial presentation was 20.0 (IQR 10.3, 32.0). Additional baseline characteristics are summarized in Table 1.

**Table 1:** Baseline characteristics of sport-related concussion patients with early and delayed symptom onset.

	**Total**	**DSO**	**ESO**	***P* value^a^**
	***N* = 144**	***N* = 24 (16.7%)**	***N* = 120 (83.3%)**	
Mean age (SD)	14.6 (1.8)	14.2 (2.0)	14.68 (1.7)	0.24^*^
Male	88 (61.1%)	15 (62.5%)	73 (60.8%)	0.88
Median days from injury to medical assessment (IQR)	5.0 (2.0, 17.4)	6.5 (2.0, 15.5)	5.0 (2.0, 19.0)	0.52^‡^
Median PCSS score (IQR) at initial medical assessment	20.0 (10.3, 32.0)	18.0 (8.0, 26.0)	20.0 (11.0, 32.8)	0.35^‡^
Loss of consciousness	14 (9.7%)	0 (0.0%)	14 (11.7%)	0.13^§^
Post-traumatic amnesia	34 (23.6%)	3 (12.5%)	31 (25.8%)	0.20^†^
History of previous concussion	58 (40.3%)	9 (37.5%)	49 (40.8%)	0.76^†^
History of headache or migraine	10 (6.9%)	1 (4.2%)	9 (7.50%)	1.00^§^
History of a learning disorder or ADHD	6 (4.2%)	1 (4.2%)	5 (4.2%)	1.00^§^
History of depression	4 (2.8%)	0 (0.0%)	4 (3.3%)	1.00^§^
Family history of mental health disorder	34 (23.6%)	4 (16.7%)	30 (25.0%)	0.43^§^
Concussions occurring during a game^a^	116 (80.6%)	19 (79.2%)	97 (80.8%)	0.85
Concussions occurring during a contact sport^b^	137 (95.1%)	23 (95.8%)	114 (95.0%)	1.00^†^
**SPORT PLAYED AT TIME OF CONCUSSION**				
Hockey	61 (42.4%)	10 (41.7%)	51 (42.5%)	
Football	24 (16.7%)	4 (8.3%)	20 (16.7%)	
Soccer	18 (12.5%)	4 (8.3%)	14 (11.7%)	
Basketball	11 (7.6%)	0 (0.0%)	11 (9.2%)	
Other^c^	30 (20.8%)	6 (25.0%)	24 (20.0%)	

a Comparison between ESO versus DSO (

* *Two sample t-test*;

† *Chi-square test*;

‡ *Two sample Mann-Whitney rank sum*;

§ *Fishers exact test)*.

b *Compared to concussions that occurred during an organized practice or a supervised gym class*.

c *Contact sports: basketball, dodgeball, football, hockey, judo, ringette, rugby, soccer, volleyball, waterpolo, and wrestling*.

*Non-contact sports: dance, gymnastics, snowboarding, and speedskating*.

*SD, standard deviation; IQR, interquartile range; ADHD, attention-deficit-hyperactivity disorder; PCSS, Post-Concussion Symptom Scale; ESO, early symptom onset; DSO, delayed symptom onset*.

Among these patients, 120 (83.3%) reported experiencing ESO while 24 (16.7%) experienced DSO following injury. Among those who experienced DSO the median length of time from the suspected injury to symptom onset was 60.0 min (IQR 20.0, 720.0). Sports-related concussion patients who experienced DSO underwent initial medical assessment at the concussion program a median of 6.5 days (IQR 2.0, 15.5) post-injury while those with ESO were assessed a median of 5.0 days (IQR 2.0, 19.0) post-injury (*p* = 0.52). No significant differences were observed between those with ESO and DSO when considering baseline characteristics including age, sex, personal and family medical histories, setting of sport, and sport played at the time of concussion. There were no significant differences in median symptom severity at initial medical assessment between those with ESO vs. DSO (20.0 vs. 18.0, *p* = 0.35).

At the end of the study period, 115 (79.9%) patients achieved physician-documented clinical recovery, 26 (18.1%) were lost to follow-up, and 3 (2.1%) remained in treatment. The median length of physician-documented recovery for the entire cohort was 22.0 days (IQR: 16.0, 34.0). Among those who achieved physician-documented clinical recovery, 41 (35.7%) experienced delayed physician-documented clinical recovery and symptom onset was not associated with the development of delayed physician-documented clinical recovery (ESO: 34.4 vs. DSO: 42.1%, *p* = 0.52). There was also no significant difference in days until physician-documented recovery among those who experienced ESO or DSO (22.0 vs. 24.0 days; *p* = 0.46) (Table 2).

**Table 2:** Symptom severity and recovery outcomes in sport-related concussion patients with early and delayed symptom onset.

	**Total**	**DSO**	**ESO**	***P* value^a^**
	***N* = 115**	***N* = 19 (16.7%)**	***N* = 96 (83.3%)**	
Days to physician documented clinical recovery (median, IQR)	22.0 (16.0, 34.0)	24.0 (17.0, 32.0)	22.0 (15.0, 34.0)	0.465^*^
Experienced delayed physician diagnosed delayed recovery (>28 days)	41 (35.7%)	8 (42.1%)	33 (34.4%)	0.520^†^

a Comparison between ESO vs. DSO (

* *Two sample Mann-Whitney rank sum*;

† *Chi-square test)*.

*Analysis completed for patients with complete medical follow-up*.

*IQR, interquartile range; PCSS, Post-Concussion Symptom Scale; ESO, early symptom onset; DSO, delayed symptom onset*.

Overall, 26 participants (18.1%) were lost to follow-up (DSO 5, ESO = 21); however, the proportion of lost to follow-up did not significantly differ between the two groups (*p* = 0.70). The only significant differences in baseline characteristics was significantly higher proportion of family history of psychiatric disorders among those lost to follow-up (*p* = 0.049) and significantly longer time between injury and initial consult (*p* = 0.04). A sensitivity analysis was conducted where the length of time from injury to medical clearance for those who received a physician diagnosed medical clearance was combined with the length of time from injury to the last appointment date for those who were lost to follow-up. Those with DSO were medically cleared or attended their last follow-up appointment at a median of 23.0 days (IQR: 17.0, 32.0) after their injury, and those with ESO were medically cleared or attended their last follow-up appointment at a median of 21.0 days (IQR: 15.0, 32.0) following injury (*p* = 0.53).

**Figure 1:**
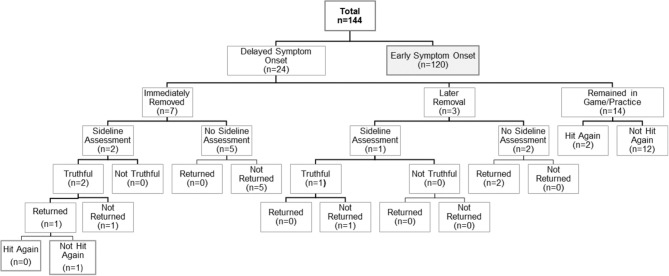
Summary of self-reported sideline management among sport-related concussion patients with delayed symptom onset.

**Figure 2:**
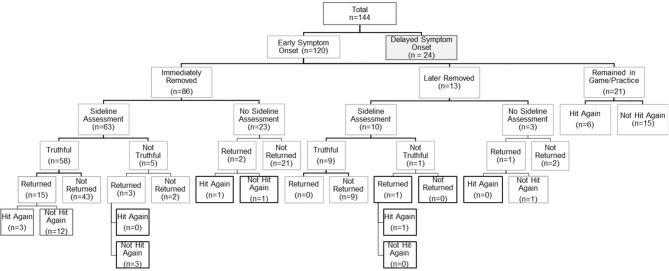
Summary of self-reported sideline management among sport-related concussion patients with early symptom onset.

At the time of suspected injury, 86 (71.6%) of athletes who experienced ESO underwent immediate removal from play, 13 (10.8%) underwent delayed removal and 21 (17.5%) remained in the same game or practice. Among those who experienced DSO, 7 (29.2%) underwent immediate removal, 3 (12.5%) underwent delayed removal and 14 (58.3%) remained in the same game or practice after the time of the suspected injury. Overall, SRC patients who reported experiencing ESO were significantly more likely to be immediately removed from play compared to those who experienced DSO (71.6 vs. 29.2%; *p* < 0.0001). Additional data regarding sideline assessment and management for this cohort is presented in Figures 1, 2.

## Discussion

This study provides important insight into the heterogeneity in symptom onset following pediatric SRC and the relationship between delayed symptom onset and clinical outcomes and athlete sideline management.

In this prospective study of pediatric SRC patients who were evaluated at a multi-disciplinary concussion program a median of 5 days post-injury, 16.7% reported experiencing delayed onset of post-concussion symptoms following injury. Among those who experienced DSO, the median duration of time from the suspected concussive injury to symptom onset was 60 min. Despite evaluating a number of baseline demographic and clinical variables, no specific risk factors for DSO were identified. Although patients who experienced DSO underwent initial medical assessment at the concussion program a median of 1.5 days later than those with ESO, no significant differences in initial symptom burden were observed between the two groups. Overall, there were no significant differences in the length of physician-documented recovery or the proportion of patients who experienced delayed physician-documented clinical recovery among those with ESO and DSO.

Although SRC has been historically viewed as a condition that typically results in the rapid onset of short-lived neurological impairment following the transmission of biomechanical forces to the head (1), limited studies have examined the onset of symptoms following this injury and its effect on clinical outcomes. In a study of collegiate athletes, Duhaime et al. (10) found that symptom onset among 44 acute concussions was immediate in 54%, delayed in 25% (no standardized definition) and unknown in 20%. In a previous retrospective study of SRC patients presenting to our multi-disciplinary pediatric concussion program, 28.1% of acute SRC patients reported that the majority of their symptoms started more than 1 h post-injury (11). In addition, Morgan et al. (12) performed a retrospective case-control study of pediatric SRC patients evaluated at a tertiary sport concussion clinic who recovered within 3 weeks post-injury and those that developed persistent symptoms lasting >3 months. They found that 2.5% of patients who recovered within 3 weeks and 25% of patients who developed persistent symptoms experienced delayed symptom onset (symptom onset >3 h post-injury), and that delayed symptom onset was associated with a greater odds of developing persistent symptoms lasting >3 months.

In addition to examining the effect of symptom onset on traditional clinical outcomes such as initial symptom burden, length of recovery, and the risk of developing delayed recovery, this study also provides important insight into how symptom onset may complicate the sideline assessment and management of children and adolescents with suspected acute SRC. Current international and national guidelines recommend that youth athletes with a suspected concussion be immediately removed from play and undergo sideline assessment by a licensed healthcare professional (1, 5–7). In instances where a licensed healthcare provider is not available, urgent referral to a physician for medical assessment is recommended (1). Although sideline tools such as the ChildSCAT5 and SCAT5 contain objective measures of cognitive and balance functioning that demonstrate some sensitivity to acute concussion (21), experts warn that these tests may be normal in the setting of acute concussion, leaving sideline healthcare professionals and sport stakeholders to rely heavily on the athlete's recognition and reporting of their concussion-like symptoms to determine the appropriate management of the athlete. Previous studies suggest that athletes who undergo delayed removal from play, following a symptomatic SRC, experience more severe symptoms and take longer to achieve clinical recovery (8, 9). In instances where an athlete has sustained an injury that is sufficient to cause a concussion but results in DSO, there is a similar risk that the athlete will remain in play or be returned to play in an asymptomatic state which may be associated with increased vulnerability to additional injury and could lead to more severe or prolonged symptoms as well as more severe forms of traumatic brain injury (3, 4). In a recent study of collegiate athletes, Asken et al. (13) found that those athletes who were immediately removed from their sporting activity reported less severe initial concussion symptoms, experienced a shorter duration of symptoms, and were at lower risk of taking >14 and >21 days to recovery from their injury. Among this cohort, 36% reported experiencing DSO (no standardized definition of delayed symptoms was provided). Although a significantly higher proportion of athletes who underwent delayed removal from their sporting activity were found to experience delayed (48.1%) vs. immediate (15.1%) symptom onset, there was no main effect of symptom onset or interaction between symptom onset and removal from sport status on recovery outcomes. In the present study, SRC patients who experienced ESO were significantly more likely to be immediately removed from play compared to those who experienced DSO; however, the impact of removal status on patient outcomes was difficult to assess in this sample given the heterogeneity in sideline assessment and management reported by these athletes. Indeed a significant proportion of athletes included in this study reported experiencing concussion symptoms but were not removed from play while others who did report experiencing concussion symptoms and were evaluated on the sidelines were also returned to play.

The results of this study have important implications for concussion education and public health policy in Canada. These findings suggest that an important proportion of youth athletes who sustain acute concussion experience DSO, which can lead to challenges in early identification and removal of these athletes from sports. This study also suggests that a notable proportion of youth athletes with suspected concussion are being allowed to return to the same sport activity where they are at risk of being exposed to additional and potentially life-threatening brain injuries. To address these knowledge gaps in Canada, Parachute has partnered with the Public Health Agency of Canada and national leaders in sport to develop the *Canadian Guideline on Concussion in Sport* (7). This guideline recommends that all sport stakeholders including athletes, parents, coaches, teachers, and officials review a standardized *Pre-season Concussion Education Sheet* that provides information on the signs and symptoms of concussion, indicates that concussion symptoms can develop immediately and in a delayed fashion following injury, and recommends that all youth with a suspected concussion be immediately and permanently removed from play until they undergo medical assessment regardless of the results of sideline assessment. In those athletes who sustain an injury but do not report symptoms or demonstrate signs of concussion, the guideline recommends that the athlete may be returned to play but should be followed for delayed symptom. Since the release of this guideline in 2017, Sport Manitoba and other community leaders have begun work to develop harmonized sport-specific concussion protocols for school divisions and youth sport organizations throughout the province that encourage all sport stakeholders to undergo pre-season education using this national resource (22–24). However, at the time of the present study there were no provincial sport- or school protocols mandating pre-season concussion education for youth sports, which may have contributed to the inconsistent and sub-optimal sideline management that some athletes received during this study period.

This study has several important limitations. First, much of the collected data included in this study was based on patient self-report and therefore is subject to recall bias and can also be impacted by post-traumatic amnesia in some instances. Data on symptom onset and sideline care depended entirely on accurate recognition and reporting by patients as well as collateral history provided by parents. Patient impressions regarding what constituted a sideline assessment or an unstructured encounter with a coach or parent may have differed between study subjects. Given that all patients were in the presence of one of their parents during initial assessment by the physician and structured interview by the research assistant, this may have influenced how patients reported the events of their injury. This is a common limitation of concussion research and without obtaining collateral history from sideline sport stakeholders (i.e., team trainers or coaches) or utilizing technology such as video replay, it is impossible to create an accurate reconstruction of injury events and sideline management among this cohort. Second, 18.1% of participants were lost to follow-up. This limitation is common among those conducted at tertiary concussion clinics; however, a sensitivity analysis revealed that the baseline characteristics among those lost and not lost to follow-up were similar with the exception of those who were lost to follow-up were more likely to have family history of psychiatric disorders. Timing of symptom onset was not associated with being lost to follow-up. Finally, study participants were recruited from a multi-disciplinary pediatric concussion program and therefore may represent a patient population with more severe injuries. It is possible that the proportion of youth with DSO vs. ESO in this study may not be reflective of the general pediatric SRC population; however, these results are likely an accurate reflection of SRC patients evaluated at tertiary concussion clinics.

In conclusion, this prospective study confirms that children and adolescents who suffer an SRC can experience delayed onset of self-reported concussion symptoms following injury. Although SRC patients who experienced DSO were less likely to be immediately removed from play, we observed no significant differences in symptom burden at initial assessment, length of physician-documented clinical recovery, or the development of delayed physician-documented clinical recovery. The sideline care of youth athletes with suspected concussion would benefit from the development of accurate and reliable sideline diagnostic tools that do not rely on athlete symptom reporting and can be administered in sport settings with limited access to sideline healthcare professionals. Until such tests are available, additional work is needed to improve concussion education among all sport stakeholders and reinforce the message that all youth athletes with a suspected concussion during sport or recreational activities should be immediately and permanently removed from play until they undergo medical assessment.

## Data Availability Statement

The datasets for this article are not publicly available because the participants did not consent to their data being publically available. Requests to access the datasets should be directed to Shelly Rempel-Rossum at Shelly.rempel-rossum@umanitoba.ca.

## Ethics Statement

This study was carried out in accordance with the recommendations of the Research Ethics Board Guidelines, University of Manitoba Bannatyne Campus Research Ethics Board with written informed consent from all subjects. All subjects gave written consent in accordance with the Declaration of Helsinki. The protocol was approved by the University of Manitoba Bannatyne Campus Research Ethics Board.

## Author Contributions

KR and ME conceptualized and designed the study, carried out the data collection and analysis, drafted the initial manuscript, critically reviewed and revised the manuscript, and approved the final manuscript as submitted. AO and ES carried out data collection and analysis, critically reviewed and revised the manuscript, and approved the final manuscript as submitted. All authors agree to be accountable for all aspects of the work ensuring that questions related to the accuracy and integrity of any part of the work are appropriately investigated and resolved.

### Conflict of Interest

The authors declare that the research was conducted in the absence of any commercial or financial relationships that could be construed as a potential conflict of interest.
